# A Genome Wide Association Study Identifies Common Variants Associated with Lipid Levels in the Chinese Population

**DOI:** 10.1371/journal.pone.0082420

**Published:** 2013-12-30

**Authors:** Li Zhou, Meian He, Zengnan Mo, Chen Wu, Handong Yang, Dianke Yu, Xiaobo Yang, Xiaomin Zhang, Yiqin Wang, Jielin Sun, Yong Gao, Aihua Tan, Yunfeng He, Haiying Zhang, Xue Qin, Jingwen Zhu, Huaixing Li, Xu Lin, Jiang Zhu, Xinwen Min, Mingjian Lang, Dongfeng Li, Kan Zhai, Jiang Chang, Wen Tan, Jing Yuan, Weihong Chen, Youjie Wang, Sheng Wei, Xiaoping Miao, Feng Wang, Weimin Fang, Yuan Liang, Qifei Deng, Xiayun Dai, Dafeng Lin, Suli Huang, Huan Guo, S. Lilly Zheng, Jianfeng Xu, Dongxin Lin, Frank B. Hu, Tangchun Wu

**Affiliations:** 1 MOE Key Lab of Environment and Health, School of Public Health, Tongji Medical College, Huazhong University of Science & Technology, Wuhan, Hubei, China; 2 Department of Epidemiology, School of Public Health and Management, Chongqing Medical University, Chongqing, China; 3 Institute of Urology and Nephrology, First Affiliated Hospital & Center for Genomic and Personalized Medicine, Guangxi Medical University, Nanning, Guangxi, China; 4 State Key Laboratory of Molecular Oncology, Cancer Institute & Hospital, Chinese Academy of Medical Sciences and Peking Union Medical College, Beijing, China; 5 Dongfeng Central Hospital, Dongfeng Motor Corporation and Hubei University of Medicine, Shiyan, Hubei, China; 6 Departments of Nutrition and Epidemiology, Harvard School of Public Health, Boston, Massachusetts, United States of America; 7 Key Laboratory of Nutrition and Metabolism, Institute for Nutritional Sciences, Shanghai Institutes for Biological Sciences, Chinese Academy of Sciences, Graduate School of the Chinese Academy of Sciences, Shanghai, China; 8 Fudan University Institute of Urology, Huashan Hospital, & Fudan-VARI Center for Genetic Epidemiology, School of Life Sciences, Fudan University, Shanghai, China; Innsbruck Medical University, Austria

## Abstract

Plasma lipid levels are important risk factors for cardiovascular disease and are influenced by genetic and environmental factors. Recent genome wide association studies (GWAS) have identified several lipid-associated loci, but these loci have been identified primarily in European populations. In order to identify genetic markers for lipid levels in a Chinese population and analyze the heterogeneity between Europeans and Asians, especially Chinese, we performed a meta-analysis of two genome wide association studies on four common lipid traits including total cholesterol (TC), triglycerides (TG), low-density lipoprotein cholesterol (LDL) and high-density lipoprotein cholesterol (HDL) in a Han Chinese population totaling 3,451 healthy subjects. Replication was performed in an additional 8,830 subjects of Han Chinese ethnicity. We replicated eight loci associated with lipid levels previously reported in a European population. The loci genome wide significantly associated with TC were near *DOCK7*, *HMGCR* and *ABO*; those genome wide significantly associated with TG were near *APOA1/C3/A4/A5* and *LPL*; those genome wide significantly associated with LDL were near *HMGCR*, *ABO* and *TOMM40*; and those genome wide significantly associated with HDL were near *LPL*, *LIPC* and *CETP*. In addition, an additive genotype score of eight SNPs representing the eight loci that were found to be associated with lipid levels was associated with higher TC, TG and LDL levels (*P* = 5.52×10^-16^, 1.38×10^-6^ and 5.59×10^-9^, respectively). These findings suggest the cumulative effects of multiple genetic loci on plasma lipid levels. Comparisons with previous GWAS of lipids highlight heterogeneity in allele frequency and in effect size for some loci between Chinese and European populations. The results from our GWAS provided comprehensive and convincing evidence of the genetic determinants of plasma lipid levels in a Chinese population.

## Introduction

Plasma lipid levels are well-established risk factors for cardiovascular disease [1–3]. High levels of total cholesterol (TC), triglycerides (TG) and low-density lipoprotein cholesterol (LDL) are associated with increased risk of cardiovascular disease, whereas high levels of high-density lipoprotein cholesterol (HDL) are associated with decreased risk of cardiovascular disease. Abnormal lipid levels are common reasons for clinical therapeutics and preventative measures. The levels of lipids in plasma are highly heritable suggesting an important role for genetic factors. Recent genome wide association studies (GWAS) have identified several loci and single nucleotide polymorphisms (SNPs) that are associated with lipid levels in European populations [4–6]. Teslovich et al. reported 95 significantly lipid-associated loci in >100,000 individuals of European ancestry and observed that most loci have the same direction of effect in Europeans and in East Asians, although the vast majority of loci do not achieve genome wide significance in the much smaller sample of East Asians (N~15,000) [6]. A GWAS conducted in a Japanese population for lipid traits replicated 6 loci (including *CETP*, *LIPC*, *APOA5* cluster, *LPL*, *GCKR* and *DOCK7-ANGPTL3*) that were associated with HDL and TG levels [7]. Their comparison with reports from GWAS in European populations provided evidence that the genetic variation has different magnitudes of effect in the different ethnicities. Further work is required to confirm the lipid-associated loci and their effects, particularly in other ethnic populations. 

Until now no GWAS has comprehensively investigated the genetic determinants of plasma lipid levels in a Chinese population. Therefore, the goal of the present study was to identify the genetic factors influencing four common lipids including total cholesterol (TC), triglycerides (TG), low-density lipoprotein cholesterol (LDL) and high-density lipoprotein cholesterol (HDL) by using a meta-analysis of two GWAS with a total 3,451 subjects in a Han Chinese population. A replication study consisting of 8,830 independent healthy subjects was used to replicate the promising SNPs in the discovery stage of the GWAS. We examined heterogeneity in allele frequency and effect size for 37 loci between Chinese and European populations. We also conducted stratified analysis for the lipid-associated SNPs to test whether the environmental factors (alcohol consumption and smoking) influenced lipid levels. Furthermore, we calculated a genotype score to assess the cumulative effects of genetic loci on lipid levels. 

## Results

 The general characteristics and plasma lipid levels of the participants were presented in Table 1. Together, we carried out two GWAS on four lipid traits TC, TG, LDL and HDL in the DFTJ-cohort (1,452 subjects) and the FAMHES (1,999 subjects) and meta-analysis of these two GWAS. Replication was performed in additional 8,830 subjects. All participants were of Han Chinese ethnicity.

**Table 1:** Characteristics of the subjects who participated in this study.

**Variables**	**DFTJ-GWAS (n=1,452)**	**FAMHES-GWAS (n =1,999)**	**DFTJ-replication (n=8,830)**
Age, mean (S.D.)	63.0 (8.1)	37.5 (11.1)	61.9 (7.8)
Sex, *n* (%)			
Male	1,136 (78.2)	1,999 (100)	3,689 (41.9)
Female	316 (21.8)	0 (0)	5,141 (58.2)
Body mass index, kg/m^2^	24.7 (3.3)	23.3 (3.4)	24.3 (3.3)
Smoking, *n* (%)			
Nonsmoker	708 (48.8)	984 (49.2)	6,423 (73.3)
Smoker	732 (50.4)	1,015 (50.8)	2,345 (26.7)
Drinking, *n* (%)			
Nondrinker	817 (56.3)	348 (17.4)	6,664 (75.5)
Drinker	634 (43.7)	1,651 (82.6)	2,159 (24.5)
TC (mmol/L)	5.03 (0.99)	5.70 (1.04)	5.21 (0.94)
TG (mmol/L)	1.41(1.01)	1.55 (1.79)	1.41 (0.97)
HDL (mmol/L)	1.43 (0.47)	1.41 (0.33)	1.40 (0.34)
LDL (mmol/L)	2.98 (0.83)	2.96 (0.80)	3.03 (0.80)

Data are mean values and SD for quantitative traits, absolute counts and percentage for binary traits.

 The quantile-quantile plot (Q-Q plot) revealed a good match between the distributions of the observed *P* values and those expected by chance (Figure S1), and the small genomic-control inflation factor (λ) between 1.011 and 1.029 also indicated a low possibility of false-positive associations resulting from population stratification. 

**Figure 1:**
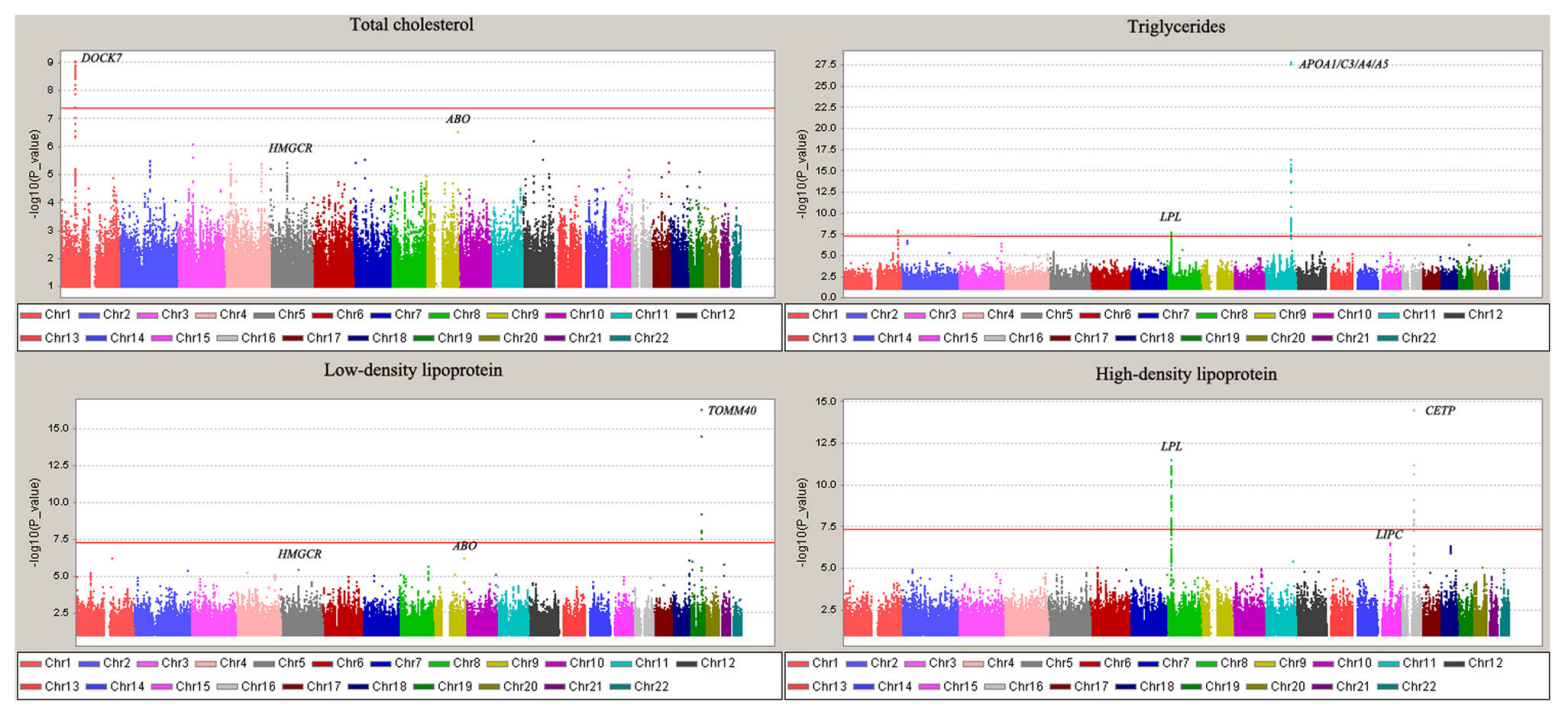
Manhattan plots of a GWAS meta-analysis of lipid levels in a total of 3,451 subjects in a Chinese population. The horizontal axis shows the chromosomal positions; the vertical axis shows –log_10_ *P* values from the linear regression. The red horizontal line represents the significance level of 5.0×10^-8^. The genes that are genome wide significant in the combined analysis are indicated with its gene names in this figure.

In the first stage meta-analysis, SNPs of 52 loci were identified with a P value lower than 10^-5^ (Figure 1). 42 of these 52 loci were not reported in previous studies. In the validation stage, we selected 63 SNPs from the 52 loci for follow up in 8,830 independent subjects (Table 2 and Table S1). When the data from the two stages were combined, 8 loci reached a genome wide significance level of 5.0×10^-8^ (combined *P* = 1.54×10^-8^ - 2.38×10^-59^, Table 2 and Figure S2). The 8 confirmed loci were *DOCK7*, *HMGCR, ABO, APOA1/C3/A4/A5, LPL, TOMM40, CETP* and *LIPC*. 

**Table 2:** Genome wide significant loci with lipid traits in a Chinese population.

						**GWAS (n=3,451)**			**Replication (n=8,830)**			**Combined (n=12,281)**
**Trait**	**SNP**	**Chr.**	**Position**	**Genes**	**Minor/ major allele**	**MAF**	**Effect size (s.e.m.)**	***P* value**	**MAF**	**Effect size (s.e.m.)**	***P* value**	***P* value**
TC	rs11207995	1	62822139	*DOCK7*	C/A	0.20	-0.032(0.005)	3.27×10^-9^	0.22	-0.012(0.006)	4.57×10^-4^	5.76×10^-10^
TC	rs10045497	5	74672239	*HMGCR*	C/A	0.50	0.025(0.007)	3.80×10^-6^	0.49	0.022(0.005)	9.89×10^-7^	1.55×10^-8^
LDL	rs10045497	5	74672239	*HMGCR*	C/A	0.50	0.096(0.019)	4.93×10^-7^	0.49	0.100(0.020)	6.37×10^-7^	1.19×10^-12^
TG	rs328	8	19864003	*LPL*	G/C	0.13	-0.114(0.020)	1.91×10^-8^	0.09	-0.071(0.023)	1.90×10^-3^	2.50×10^-10^
HDL	rs328	8	19864003	*LPL*	G/C	0.13	0.053(0.008)	9.75×10^-12^	0.09	0.040(0.010)	6.61×10^-5^	1.69×10^-14^
TC	rs507666	9	135139219	*ABO*	A/G	0.18	0.036(0.007)	2.91×10^-7^	0.23	0.015(0.003)	4.27×10^-6^	3.55×10^-11^
LDL	rs507666	9	135139219	*ABO*	A/G	0.18	0.165(0.031)	9.25×10^-8^	0.23	0.073(0.015)	5.41×10^-7^	2.10×10^-11^
TG	rs651821	11	116167788	*APOA1/C3/A4/A5*	C/T	0.27	0.162(0.015)	1.35×10^-28^	0.28	0.170(0.014)	1.01×10^-31^	2.38×10^-59^
HDL	rs2043085	15	56468245	*LIPC*	T/C	0.47	0.036(0.006)	3.02×10^-7^	0.47	0.012(0.006)	4.40×10^-2^	1.54×10^-8^
LDL	rs1160985	19	50095251	*TOMM40*	T/C	0.36	-0.111(0.025)	6.13×10^-6^	0.37	-0.127(0.022)	1.53×10^-8^	3.68×10^-13^
HDL	rs3764261	16	55550824	*CETP*	T/G	0.17	0.086(0.012)	6.65×10^-12^	0.16	0.062(0.008)	2.00×10^-15^	1.93×10^-25^

The minor allele is the effect allele and the major allele is the reference allele.

Chr., chromosome; MAF, minor allele frequency.

In this table, all loci are presented that had a genome wide significant *P* value (*P* <5.0×10^-8^) in the combined analyses of the first stage meta-analysis and the replication stage on the four lipid traits TC, TG, LDL and HDL. Furthermore, the results for the first stage meta-analysis is given in the columns titled "GWAS" and the results of the validation stage in the columns titled "Replication". For each locus the most significant SNP is reported.

For TC, we confirmed three previously reported loci. SNP rs507666 in the intron of *ABO* gene on chromosome 9q34 was associated with TC (combined *P* = 3.55×10^-11^). Additionally, we confirmed two other loci with prior evidence for association with TC levels (*DOCK7* and *HMGCR*). We found that the SNPs rs11207995 (combined *P* = 5.76×10^-10^) in *DOCK7* on chromosome 1 and rs10045497 (combined *P* = 1.55×10^-8^) in *HMGCR* on chromosome 5 showed strong association in the Han Chinese population (Table 2). For TG, we confirmed two loci with prior evidence (*LPL* and *APOA1/C3/A4/A5*). We observed that SNPs at *APOA1/C3/A4/A5* loci on chromosome 11q23.3 had the strongest association with TG levels (combined *P* = 2.38×10^-59^ for rs651821) in the Han Chinese population. The SNP rs328 in *LPL* was significantly associated with TG levels (combined *P* = 2.50×10^-10^). As for LDL, we not only validated two loci that were associated with TC levels (*HMGCR* and *ABO*), but also confirmed another region (*TOMM40*, combined *P* = 3.68×10^-13^ for rs1160985) for which there was prior evidence of association [5,8]. The SNPs rs10045497 in *HMGCR* and rs507666 in *ABO* were also associated with LDL levels, with combined *P* = 1.19×10^-12^ and 2.10×10^-11^, respectively. For HDL, we replicated findings from a previous GWAS with respect to *LPL*, *LIPC* and *CETP* loci (combined *P* = 1.69×10^-14^ for rs328 in *LPL*, 1.54×10^-8^ for rs2043085 in *LIPC*, and 1.93×10^-25^ for rs3764261 in *CETP*, respectively) (Table 2). However, we could not replicate any loci that have not been reported to be associated with lipid levels in previous studies (Table S1).

We compared the findings from the present study with reported loci and SNPs in Europeans and Japanese. We selected 47 SNPs in 37 lipid-associated loci that had been identified in at least two previous European studies to compare with. Among the 37 loci, 31 loci were also studied in a Japanese population. As shown in Table S2, *APOA1/C3/A4/A5* and *LPL* were associated with lipid levels across different populations. In addition, we found that 37 of 47 SNPs had the same direction in our study as those reported in European populations, but did not reach genome wide significance (*P* > 5 ×10^-8^) in meta-analysis of two GWAS (DFTJ and FAMHES) in discovery stage in the present study. For some SNPs that were associated in Europeans but not in Chinese populations we observed differences in allele frequency, such as rs599839 on *CELSR2-PSRC1-SORT1* loci and rs693 in *APOB*. The minor allele frequency (MAF) of rs599839 on *CELSR2-PSRC1-SORT1* loci is 0.24 in the European population, while it is only 0.06 in the Han Chinese population (Table 3). *APOB* was significantly associated with HDL levels in Europeans. The MAF of rs693 in *APOB* was also different between the Han Chinese population (MAF = 0.06) and European population (MAF = 0.48). We observed that rs693 was not significantly associated with plasma HDL levels in the Chinese population (*P* = 0.146; Table 3). We therefore evaluated the associations of another SNP rs673548 in *APOB* which had a high MAF 0.26 in Chinese population. This SNP was in linkage disequilibrium (LD) with the reported SNP rs693 in HapMap CEU data (D’=1.0, r^2^ = 0.23) and CHB data (D’=1.0, r^2^ = 0.08). In our data, rs673548 also showed no significant associations with HDL levels (*P* = 0.011; Table 3). No significant association with HDL levels of any other SNPs in the *APOB* gene was observed. Several loci that were found in analyses on European populations could not be identified in our study of Chinese individuals. This is consistent with a previous comparison of results on Europeans with analyses in a Japanese study [7]. All these results indicated that ethnic genetic differences exist among different populations. 

**Table 3:** Ethnic differences in major genetic variants associated with lipid levels.

				**Han Chinese population** ^a^				**Japanese population** ^b^				**European population** ^c^		
**Trait**	**Gene**	**SNP**	**Position**	**MAF**	**LD (r^2^)**	***P***		**MAF**	**LD (r^2^)**	***P***		**MAF**	**LD (r^2^)**	***P***
**LDL**	***CELSR2-PSRC1-SORT1* (1p13.3)**	rs599839	109623689	0.06	ref	0.015		0.08	ref	NA		0.24	ref	3×10^-21^
		rs646776	109620053	0.04	0.33	1.23×10^-3^		0.07	0.85	1.30×10^-3^		0.24	0.89	3×10^-29^
		rs12740374	109619113	0.04	0.33	9.31×10^-4^		0.07	0.85	NA		0.21	0.89	2×10^-42^
**HDL**	***APOB* (2p24.1)**	rs673548	21091049	0.26	ref	0.011		0.36	ref	NA		0.24	ref	7.4×10^-7^
		rs693	21085700	0.06	0.08	0.146		0.09	0.18	NA		0.48	0.23	1.3×10^-7^
		rs6754295	21059688	0.29	0.85	0.145		0.29	0.72	0.355		0.25	0.86	4.4×10^-8^

MAF, minor allele frequency; LD, linkage disequilibrium; NA: data not available.

^a^ The P values in a Han Chinese population were from meta-analysis of two GWAS (DFTJ-cohort and FAMHES) in discovery stage.

^b^ The P values in a Japanese population were cited from Reference (7).

^c^ The P values in a European population were cited from Reference (4–6).

We conducted stratified analysis for the lipid-associated SNPs to test whether alcohol consumption or smoking influenced lipid levels. We tested all eight SNPs in Table 2 and found that rs10045497 in *HMGCR* was significantly associated with TC and LDL levels in non-drinkers (*P* = 7.43×10^-9^ and 2.60×10^-8^, respectively), while no association was observed in drinkers (*P* > 0.05) (Table 4). None of the other seven SNPs showed a statistical significance with alcohol consumption or smoking (*P* > 0.05).

**Table 4:** Results of the stratification concerning alcohol intake for association of rs10045497 and lipid levels in the Chinese cohort *HMGCR*.

		**Genotypes**			
		***AA***	***AC***	***CC***	***P***
**TC** (mmol/L)	Non-drinkers	5.12±0.04	5.26±0.03	5.42±0.04	7.43×10^-9^
		(N=1472)	(N=3113)	(N=1455)	
	Drinkers	5.05±0.07	5.15±0.05	5.05±0.07	0.921
		(N=536)	(N=959)	(N=456)	
**LDL** (mmol/L)	Non-drinkers	2.91±0.03	3.02±0.02	3.16±0.03	2.60×10^-8^
		(N=1472)	(N=3113)	(N=1455)	
	Drinkers	2.91±0.06	2.99±0.04	2.96±0.06	0.593
		(N=536)	(N=959)	(N=456)	

In this table the mean and SD per trait is given stratified for drinking status and genotype. Moreover, the P values for SNP effect calculated in a multiple linear regression model adjusted for age sex and BMI are presented.

**Figure 2:**
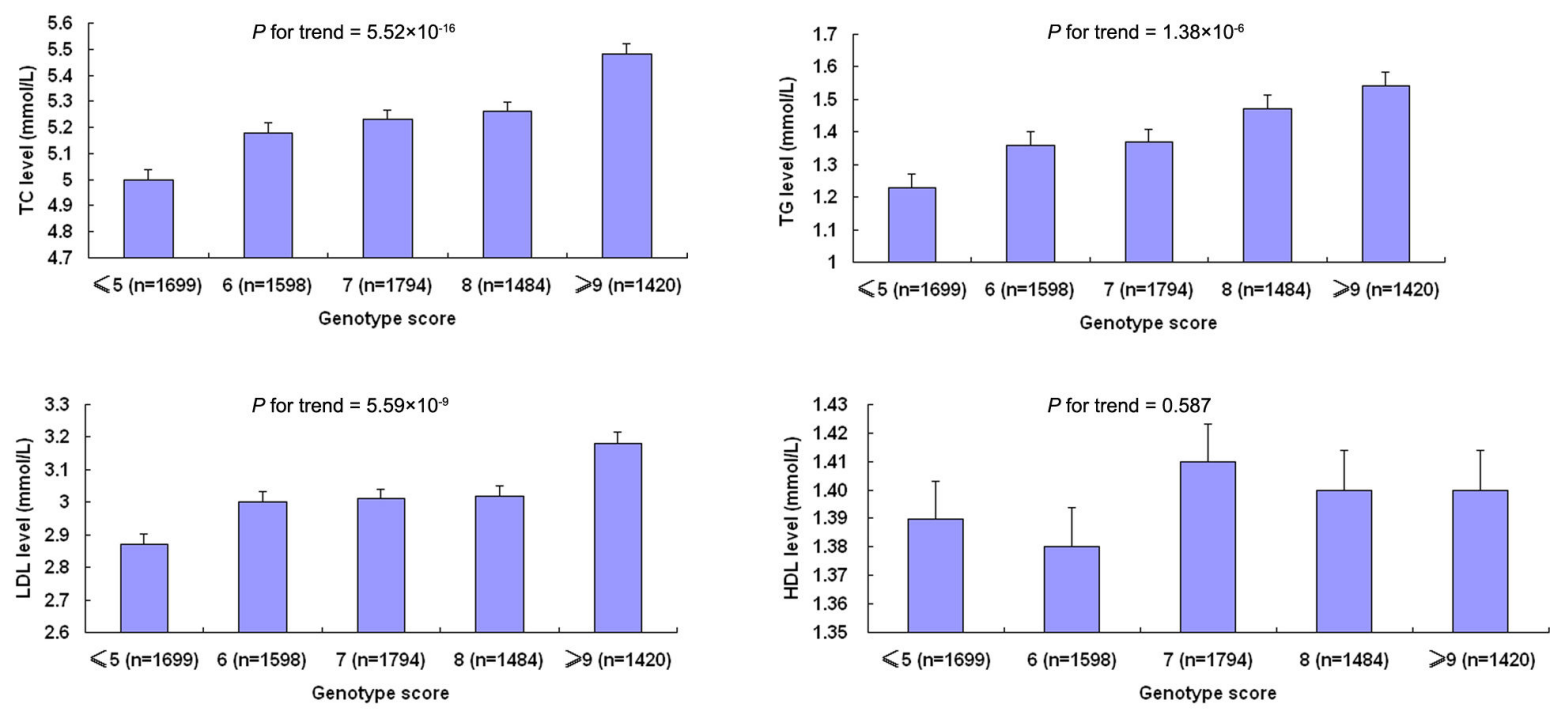
Genotype score of top SNPs with TC, TG, LDL, HDL levels. The genotype score represents the number of risk alleles (the alleles associated with higher TC or TG or LDL or lower HDL) at each of eight SNPs. The eight SNPs were rs11207995 (DOCK7), rs10045497 (HMGCR), rs507666 (ABO), rs328 (LPL), rs651821 (APOA1/C3/A4/A5), rs1160985 (TOMM40), rs2043085 (LIPC) and rs3764261 (CETP). The height of the bars is the mean values of individuals with a specific genotype score. The error bars are Means ± SD of lipid levels in each group.

To assess the cumulative effects of genetic factors on lipid levels, we constructed a genotype score by using SNPs from the eight loci presented in Table 2 that were genome wide significant in our combined meta-analysis. The genotype score represented the number of risk alleles (the alleles associated with higher TC or TG or LDL or lower HDL) at each of the eight SNPs. As shown in Figure 2, the genotype score was associated with higher TC, TG and LDL levels. Compared with participants with a genotype score lower than 6, the TC levels in those with 9 or more genotype scores increased from 5.00 to 5.48 mmol/L (*P* = 5.52×10^-16^), the level of TG increased from 1.23 to 1.54 mmol/L (*P* = 1.38×10^-6^), the level of LDL increased from 2.87 to 3.18 mmol/L (*P* = 5.59×10^-9^), whereas the HDL level did not significantly decrease (from 1. 39 to 1.41 mmol/litre, *P* = 0.587). 

## Discussion

 In this GWAS, we have replicated eight reported loci associated with 4 lipid traits in a Han Chinese population. These observations suggest that the present study design is capable of identifying significant SNPs associated with lipid levels in the Han Chinese population. However, all loci that had a P value lower than 5×10^-8^ in the combined analyses of the first stage meta-analyses and the replication stage were previously reported. Therefore, we discussed them as confirmed loci in the following. Ethnic differences were found between Asian and European populations. Kathiresan et al. reported 30 loci [5], Aulchenko et al. reported 22 loci [9], Chasman et al. reported 43 loci [8] and Teslovich et al. reported 95 loci [6] that were significantly associated with lipid levels in European populations. Of the 95 loci, 59 were reported firstly by Teslovich et al [6]. The frequencies of minor alleles were different in European and Han Chinese populations, suggesting that genetic heterogeneity may contribute to the discrepancy between our results and previous findings in European populations. Nonetheless, we observed that 37 of 47 lead SNPs reported in European populations had the same direction of association as those in Chinese Han population, which was consistent with Teslovich et al [6]. It should be noted that the power of our analysis may not be sufficient to detect associated SNPs with low MAF or small effect size on lipid levels in the Han Chinese population. We used the Quanto software to do the statistical power calculation based on the effect size (beta estimate), our study has 99% power to detect the association of SNP with lipid levels at the 5×10^-8^ significance level (MAF= 0.10, beta = 0.15). However, the power is only 41% when beta equals to 0.10 [10]. In addition, the causal variants may not have been identified and genotyped yet, and they may be different in European and Chinese populations. Although the effect of each SNP on lipid levels was only moderate, the SNPs had a strong cumulative association with the traits when we combined the lipid-associated SNPs using a genotype score. Our findings suggested that the cumulative effect of multiple genetic loci contributed to lipid levels, which was consistent with the finding reported by Teslovich et al [6].

Some of the loci that are associated with lipid levels include obvious functional candidate genes. For example, the genes including *LPL*, *APOA5*, *LIPC* and *CETP* reported in the previous and present studies were involved in lipid metabolism and coronary artery disease (CAD). Waterworth et al. combined 8 GWAS and found that SNPs showing strong association with lipid levels at *APOB* and *APOE* loci were also associated with CAD risk [11]. We also identified 2 SNPs (rs599839 and rs16996148) that associated with lipid levels in European were significantly associated with CAD susceptibility in the Chinese Han population [12]. 

Our GWAS not only replicated the findings of the genetic variants that were associated with lipid levels in the Chinese, but also produced evidence of the effects of environmental factors on lipid levels. Our results showed that rs10045497 in *HMGCR* was significantly associated with TC and LDL levels in nondrinkers, while no association was observed in drinkers. *HMGCR* encodes 3-hydroxy-3-methylglutaryl-coenzyme A reductase (HMGCR), which is the rate limiting enzyme in cholesterol synthesis. The inhibitors of HMGCR have been used to control cholesterol levels [13]. The AA genotype of rs10045497 in *HMGCR* decreased the TC and LDL levels in nondrinkers. However, the AA genotype of rs10045497 had no protective effect when susceptible individuals were drinkers. Low levels of TC and LDL are associated with decreased risk of cardiovascular disease, and no drinking can reduce the risk of cardiovascular disease. It is inferred in our study that the positive effect of *HMGCR* genotype on lipid levels might be partly dependent on alcohol intake. Our results showed that individuals who carry AA genotype can benefit from the no alcohol intake. Tan et al identified an interaction between alcohol consumption and the *ALDH2* rs671 on TG levels [14]. These findings suggest that lipid levels might be attributed to genetic variations of multiple genes and to their interaction with an individual’s environment or lifestyle.

In summary, this study replicated the associations of eight loci with lipid levels in a Han Chinese population. Comparisons with previous GWAS of lipid levels suggested a heterogeneity in allele frequency and in effect size for some loci between Chinese and European populations. Further studies using large samples in diverse ethnic populations will be required to provide a more comprehensive understanding of the global genetics of lipid levels.

## Materials and Methods

### Ethics Statement

All participants provided written informed consent and the ethical committees of Tongji Medical College and Guangxi Medical University approved this research project.

### Subjects

In the present study, we performed a meta-analysis of two GWAS in the Han Chinese population including the Dongfeng-Tongji cohort study (DFTJ-cohort) and the Guangxi Fangchenggang Area Male Health and Examination Survey (FAMHES). In the DFTJ-cohort, we collected fasting blood samples and detailed information on demographic and lifestyle factors. The GWAS was conducted in the DFTJ-cohort among 1,461 individuals. After stringent quality control, 1,452 subjects were included in the analysis. The 8,830 healthy subjects included in the replication stage were selected from the DFTJ-cohort [15]. The FAMHES project was initiated in Fangchenggang city, Guangxi, southwestern China in 2009, including 4,303 Chinese men age 17 to 88 years old [16]. For this study, we included only those aged 20 to 69 years old who reported Han ethnicity (n = 2,012). After stringent quality control, 1,999 subjects were included in the analysis. 

The participants in both the discovery GWAS stage and the replication stage were from the general population and were healthy subjects recruited during routine health check-ups and who had no diagnosed chronic diseases such as cardiovascular disease and cancer, which minimized the potential for confounding and selection bias. In addition, detailed epidemiological characteristics including age, gender, BMI, smoking, and drinking were adjusted for in the discovery and replication stages. Subjects were classified as smokers and nonsmokers. Those who had smoked less than 100 cigarettes in their lifetimes were defined as nonsmokers; otherwise, they were defined as smokers. Alcohol consumption was classified into two categories: drinkers and nondrinkers. Those who reported drinking any alcoholic drink more often than ‘less than once a year’ or ‘never’ were defined as drinkers; otherwise, they were defined as nondrinkers. The demographic and clinical information on these subjects is summarized in Table 1.

### Determination of Lipid Levels

Blood specimens were obtained after participants had fasted overnight (≥8 h). In the DFTJ-cohort, the plasma TC, TG, LDL and HDL levels were measured by the ARCHITECT Ci8200 automatic analyzer (ABBOTT Laboratories. Abbott Park, Illinois, U.S.A.) using the Abbott Diagnostics reagents according to the manufacture’s instructions. In the FAMHES study, the plasma TC, TG, LDL and HDL levels were measured with electrochemiluminescence immunoassay on the COBAS 6000 system E601 (Elecsys module) immunoassay analyzer (Roche Diagnostics, GmbH, Mannheim, Germany), with the same batch of reagents according to the manufacture’s instructions.

### Sample genotyping

For the DFTJ-GWAS, we carried out the genotyping using Affymetrix Genome-Wide Human SNP Array 6.0 chips. For the FAMHES-GWAS, we used the Illumina Omni-Express platform to perform the genotyping. We used the iPLEX system (Sequenom, Inc., San Diego, CA, USA) to genotype the DFTJ-replication samples. Polymerase chain reaction and extension primers were designed using Mass ARRAY Assay Design 3.1 software (Sequenom, Inc.). Genotyping procedures were performed according to the manufacturer’s iPLEX Application Guide (Sequenom, Inc.). All genotyping reactions were performed in 384-well plates. Each plate included four randomly selected duplicates, as well as six negative controls using double distilled water. The genotype calling algorithms CRLMM was used for the Affymetrix Genome-Wide Human SNP Array 6.0. Genotype calls were generated with the GenCall algorithm implemented in the Illumina GenomeStudio software for the llumina Omni-Express chip. The average concordance rate for genotypes was 99.8%. The primer sequence was available upon request.

### Quality control

For the DFTJ-GWAS, we genotyped a total of 906,703 SNPs among 1,461 subjects in which 38,446 SNPs not mapped on autosomal chromosomes were excluded. After stringent QC filtering, SNPs with MAF < 0.01 (193,732 SNPs), Hardy-Weinberg Equilibrium (HWE) < 0.0001 (1,332 SNPs), and SNPs call rate < 95% (17,764 SNPs) were excluded. Individuals with a call rate < 95% were also not included for further analysis. Finally, 1,452 subjects with 658,288 autosomal SNPs were retained for statistical analyses, with an overall call rate of 99.68%. For the FAMHES-GWAS, genotypes for 2,012 individuals were measured and included in the data before QC. After stringent QC filtering, SNPs with MAF < 0.01 (235,761 SNPs), Hardy-Weinberg Equilibrium (HWE) < 0.001 (11,653 SNPs), and SNPs call rate < 95% (28,082 SNPs) were excluded. A total of 1,999 individuals with 709,211 autosomal SNPs passed the call rate of 95% and were included in the final statistical analysis. Population structure was evaluated by principal components analysis (PCA) using the software package EIGENSTRAT 3.0 [17]. PCA showed minimal evidence for population stratification in our study populations (Figure S3). The Q-Q plots were generated by using R 2.11.1 [18]. The regional plots were drawn using the LocusZoom [19,20].

### Imputation

We used the MACH 1.0 software [21] to impute ungenotyped SNPs using the LD information from the HapMap phase II database (CHB+JPT as reference set, 2007-08_rel22, released 2007-03-02) in the DFTJ-GWAS. The IMPUTE program was used to infer the ungenotyped SNPs in the FAMHES-GWAS [22,23]. Imputed SNPs with high genotype information content (proper info > 0.5 for IMPUTE and Rsq > 0.3 for MACH) were kept for further association analysis. In addition, those SNPs with MAF < 1%, derivation from HWE test (*P* ≤ 0.001), and failure to missingness test (GENO > 0.95) were also exclude for further analysis. Finally, a total of 2,249,917 SNPs remained in the final analysis in the FAMHES-GWAS. For the DFTJ-GWAS, 2116,099 SNPs were remained for the final analysis.

### Statistical methods

Each continuous trait was tested for normality and TG values were log-transformed. Genome wide association analysis was performed using the additive model by linear regression analysis with the PLINK 1.06 software [24,25]. The Manhattan plot of -log_10_ *P*, LD structures and haplotype block plots were generated by using Haploview (v4.1) [26,27]. We used ProbABEL software to analyze the hard genotype calls and do the association studies with imputation data [28,29] and METAL software [30,31] to perform the meta-analysis of the DFTJ-GWAS data of the 1,452 subjects and the FAMHES-GWAS data of 1,999 health subjects. For the association analyses of the GWAS scan and replication samples, adjustment for age, sex, BMI, smoking, and drinking was performed. For the GWAS analysis, the top two eigenvectors were also adjusted as covariates in the linear regression analysis to control for possible population stratification. SNPs selected for the replication were based on the following criteria: (1) SNP had *P* ≤ 1.0×10^-5^ in the meta-analysis of both GWAS in the discovery stage; (2) SNPs with the lowest *P* value were selected when multiple SNPs showed a strong LD (r^2^ ≥ 0.8); and (3) MAF ≥ 0.05. Heterogeneity among the study populations was evaluated by the *I* ^2^ statistic [32]. 

We used the fixed effect meta-analysis to combine evidence for association from the discovery samples and replication samples. A probability value cut-off of 5 ×10^-8^ was considered as a genome wide significance level in the present study.

We conducted stratified analysis for the lipid-associated SNPs to test whether environmental factors (alcohol consumption and smoking) influenced lipid levels in a total of 8,830 subjects in DFTJ-replication. The effect of genotypes and environmental factors on lipid levels was investigated using a multiple linear regression model with adjustment for age, sex and BMI.

The genotype score was calculated on the basis of 8 SNPs tagging lipid-associated genes in a total of 8,830 subjects in DFTJ-replication. Only the SNPs that gained a P <5×10^-8^ in combined analysis of the first and the second stage in our study were included to calculate the score. These loci included *DOCK7* (rs11207995), *HMGCR* (rs10045497), *ABO* (rs507666), *LPL* (rs328), *APOA1/C3/A4/A5* (rs651821), *TOMM40* (rs1160985), *LIPC* (rs2043085) and *CETP* (rs3764261). A simple count method was used to create the genotype score. We assumed that each SNP was independently and equally associated with the risk of lipid levels in an additive model. The count method was calculated by summing up the number of risk alleles for each of the SNPs producing a score out of 16 (the total number of risk alleles). The effects of genotype scores on lipid levels were assessed by multiple linear regression models with adjustment for age, sex, BMI. All data analyses were carried out using SAS 9.1.3 (SAS Institute, Cary, NC) if not stated otherwise.

## Supporting Information

Table S1 **Association of SNPs with lipid levels in GWAS and replication studies in a Chinese population.** The P values of SNPs were lower than 10^-5^ in discovery stage and higher than 1.0×10^-3^ after Bonferroni correction for 63 SNPs in validation stage.
(DOC)

Table S2 **Comparison of the results of the present study for lipid levels with previous studies in European and Japanese populations.**
(DOC)

Figure S1 **Q-Q plots for QTL analyses.** The horizontal axis shows -log_10_ transformed expected *P* values, while the vertical axis indicates -log_10_ transformed observed *P* values. The genomic inflation factor λ for each analysis is shown below each graph. Black line, all test statistics; red line, 35 previously reported loci (Table S2) excluded.
(DOC)

Figure S2 **Regional plots of associated loci with lipid levels.** The horizontal axis shows the chromosomal positions in the NCBI build 36 genome sequence.
(DOC)

Figure S3 **Principal component analysis (PCA) plot of samples in the current GWAS.** The plot presents the top two eigenvectors identified by principal component analysis using Eigenstrat software.
(DOC)
