# Supplementary material for: A Genome Wide Association Study Identifies Common Variants Associated with Lipid Levels in the Chinese Population
Source: PLoS One. 2013 Dec 30;8(12):e82420. doi: 10.1371/journal.pone.0082420 (PMC3875415; doi:10.1371/journal.pone.0082420)
Supplement: Table S1 — Association of SNPs with lipid levels in GWAS and replication studies in a Chinese population. The P values of SNPs were lower than 10-5 in discovery stage and higher than 1.0×10^-3^ after Bonferroni correction for 63 SNPs in validation stage. (DOC) [file pone.0082420.s001.doc]

**Table S1. Association of SNPs with lipid levels in GWAS and replication studies in a Chinese population.** The *P* values of SNPs were lower than 10^-5^ in discovery stage and higher than 1.0×10^-3^ after Bonferroni correction for 63 SNPs in validation stage.

| **Trait** | **SNP** | **Chr.** | **Position** | **Gene** | **Minor/major allele** | **MAF** | **GWAS ^a^** | | **Replication** | | **Combined*P* value** |
| --- | --- | --- | --- | --- | --- | --- | --- | --- | --- | --- | --- |
|  |  |  |  |  |  |  | **β** | ***P* value** | **β** | ***P* value** |  |
| **TC** |  |  |  |  |  |  |  |  |  |  |  |
|  | rs10158897 | 1 | 62685506 | *USP1* | T/C | 0.2 | -0.032 | 3.7×10^-9^ | -0.009 | 0.84 | 0.037 |
|  | rs13009827 | 2 | 128629405 | *UGCGL1* | A/G | 0.18 | 0.026 | 6.8×10^-6^ | 0.021 | 0.68 | 0.073 |
|  | rs10496667 | 2 | 128714692 | *UGCGT1* | A/G | 0.22 | 0.026 | 3.5×10^-6^ | 0.063 | 0.17 | 8.1×10^-4^ |
|  | rs11706832 | 3 | 66585670 | *LRIG1* | C/A | 0.17 | 0.028 | 2.4×10^-6^ | -0.008 | 0.87 | 0.154 |
|  | rs6549120 | 3 | 66590131 | *LRIG1* | G/A | 0.24 | 0.027 | 8.3×10^-7^ | 0.012 | 0.77 | 0.074 |
|  | rs3924177 | 4 | 26455462 | *STIM2* | C/G | 0.28 | -0.022 | 6.6×10^-6^ | -0.018 | 0.66 | 0.025 |
|  | rs997851 | 4 | 155057732 | *RNF175* | G/T | 0.48 | -0.02 | 7.9×10^-6^ | -0.043 | 0.25 | 0.112 |
|  | rs963872 | 5 | 2910541 | *C5orf38* | A/G | 0.18 | 0.026 | 6.5×10^-6^ | -0.041 | 0.38 | 0.367 |
|  | rs1548608 | 7 | 50869026 | *GRB10* | A/G | 0.25 | 0.028 | 2.8×10^-6^ | 0.029 | 0.47 | 0.045 |
|  | rs12579815 | 12 | 46834141 | *ASB8* | C/T | 0.08 | 0.049 | 6.3×10^-7^ | -0.07 | 0.31 | 0.243 |
|  | rs10862938 | 12 | 83752227 | *SLC6A15* | T/C | 0.16 | 0.033 | 2.9×10^-6^ | -0.001 | 0.73 | 0.137 |
|  | rs2698260 | 12 | 112677433 | *RBM19* | A/G | 0.46 | 0.023 | 9.8×10^-6^ | -0.021 | 0.59 | 0.354 |
|  | rs11855116 | 15 | 93059602 | *MCTP2* | A/C | 0.19 | -0.024 | 6.7×10^-6^ | -0.003 | 0.95 | 0.107 |
|  | rs11655599 | 17 | 73339995 | *TNRC6C* | C/T | 0.39 | -0.022 | 3.9×10^-6^ | -0.052 | 0.22 | 0.079 |
| **TG** |  |  |  |  |  |  |  |  |  |  |  |
|  | rs12757404 | 1 | 212696238 | *PTPN14* | A/G | 0.45 | -0.06 | 4.6×10^-6^ | -0.01 | 0.79 | 1.6×10^-6^ |
|  | rs876793 | 1 | 235918705 | *RYR2* | C/T | 0.2 | 0.1 | 1.0×10^-8^ | 0.009 | 0.85 | 3.4×10^-7^ |
|  | rs16835702 | 1 | 235975110 | *RYR2* | G/A | 0.21 | 0.092 | 2.1×10^-8^ | 0.013 | 0.78 | 2.7×10^-6^ |
|  | rs16835705 | 1 | 235975533 | *RYR2* | A/G | 0.2 | 0.089 | 5.6×10^-8^ | 0.013 | 0.77 | 4.2×10^-6^ |
|  | rs2250042 | 1 | 235998240 | *RYR2* | C/T | 0.19 | 0.073 | 7.8×10^-6^ | -0.028 | 0.56 | 0.231 |
|  | rs16838354 | 2 | 207140406 | *ADAM23* | G/T | 0.05 | 0.185 | 5.4×10^-6^ | -0.144 | 0.13 | 0.245 |
|  | rs6444307 | 3 | 189790211 | *LPP* | G/A | 0.1 | -0.128 | 4.4×10^-7^ | -0.014 | 0.83 | 7.2×10^-5^ |
|  | rs6841552 | 4 | 90696112 | *SNCA* | T/C | 0.08 | -0.117 | 7.9×10^-6^ | 0.006 | 0.93 | 0.112 |
|  | rs11737460 | 4 | 186165772 | *ACSL1* | G/A | 0.23 | 0.07 | 8.2×10^-6^ | -0.028 | 0.54 | 0.376 |
|  | rs1607566 | 5 | 23032827 | *CDH12* | C/T | 0.49 | -0.062 | 9.0×10^-6^ | 0.002 | 0.97 | 0.235 |
|  | rs7113382 | 11 | 41640833 | *RPL9P23* | T/C | 0.1 | 0.095 | 8.3×10^-6^ | -0.017 | 0.79 | 0.118 |
|  | rs7104498 | 11 | 64022819 | *SLC22A11* | A/G | 0.32 | 0.063 | 9.8×10^-6^ | -0.03 | 0.46 | 0.244 |
|  | rs7932967 | 11 | 116712874 | *CEP164* | T/G | 0.21 | 0.078 | 2.5×10^-6^ | -0.039 | 0.44 | 0.419 |
|  | rs3782886 | 12 | 110594871 | *BRAP* | C/T | 0.24 | -0.08 | 6.1×10^-6^ | 0.011 | 0.82 | 0.102 |
|  | rs671 | 12 | 110726148 | *ALDH2* | A/G | 0.24 | -0.081 | 3.4×10^-6^ | 0.014 | 0.77 | 0.223 |
|  | rs3813131 | 13 | 114108120 | *C13orf8* | A/G | 0.1 | 0.1 | 5.9×10^-6^ | -0.038 | 0.51 | 0.351 |
|  | rs1532085 | 15 | 56470657 | *LIPC* | A/G | 0.45 | 0.06 | 5.6×10^-6^ | 0.002 | 0.85 | 1.2×10^-4^ |
| **LDL** |  |  |  |  |  |  |  |  |  |  |  |
|  | rs10158897 | 1 | 62685506 | *USP1* | T/C | 0.2 | -0.112 | 3.2×10^-6^ | -0.021 | 0.65 | 8.5×10^-6^ |
|  | rs636523 | 1 | 62692595 | *DOCK7* | G/A | 0.2 | -0.114 | 2.5×10^-6^ | -0.021 | 0.65 | 5.1×10^-7^ |
|  | rs6549120 | 3 | 66590131 | *LRIG1* | G/A | 0.24 | 0.113 | 3.1×10^-6^ | -0.013 | 0.74 | 0.113 |
|  | rs9364112 | 6 | 71656139 | *B3GAT2* | T/C | 0.39 | 0.104 | 5.0×10^-6^ | -0.006 | 0.88 | 0.421 |
|  | rs568993 | 6 | 71658889 | *B3GAT2* | A/G | 0.28 | 0.113 | 6.0×10^-6^ | 0.065 | 0.19 | 1.7×10^-5^ |
|  | rs4921670 | 8 | 19652995 | *LPL* | T/C | 0.15 | -0.12 | 8.6×10^-6^ | -0.069 | 0.19 | 4.3×10^-6^ |
|  | rs17321515 | 8 | 126555590 | *TRIB1* | A/G | 0.47 | 0.05 | 3.51×10^-6^ | 0.070 | 3.88×10^-4^ | 8.10×10^-7^ |
|  | rs2275536 | 10 | 128711164 | *DOCK1* | T/C | 0.19 | 0.105 | 8.9×10^-6^ | -0.004 | 0.93 | 0.109 |
|  | rs8086634 | 18 | 72830179 | *MBP* | T/C | 0.15 | -0.12 | 9.9×10^-6^ | -0.048 | 0.41 | 6.4×10^-4^ |
|  | rs4804146 | 19 | 11117175 | *SPC24* | C/T | 0.26 | -0.111 | 5.5×10^-7^ | -0.053 | 0.2 | 7.2×10^-6^ |
| **HDL** |  |  |  |  |  |  |  |  |  |  |  |
|  | rs3135351 | 6 | 32500922 | *C6orf10* | A/C | 0.1 | 0.042 | 8.8×10^-6^ | -0.022 | 0.27 | 0.246 |
|  | rs11440 | 11 | 123522772 | *VWA5A* | G/C | 0.05 | 0.122 | 3.7×10^-6^ | 0.016 | 0.7 | 4.9×10^-5^ |
|  | rs8086057 | 18 | 45337103 | *LIPG* | C/T | 0.21 | 0.033 | 7.4×10^-7^ | -0.016 | 0.32 | 0.226 |
|  | rs10485461 | 20 | 44890921 | *EYA2* | G/A | 0.05 | -0.216 | 8.8×10^-6^ | 0.004 | 0.95 | 0.007 |

Chr., chromosome; MAF, minor allele frequency.

**^a^** Meta-analysis of two GWAS (DFTJ-cohort and FAMHES) in discovery stage.
