# Supplementary material for: A Genome Wide Association Study Identifies Common Variants Associated with Lipid Levels in the Chinese Population
Source: PLoS One. 2013 Dec 30;8(12):e82420. doi: 10.1371/journal.pone.0082420 (PMC3875415; doi:10.1371/journal.pone.0082420)
Supplement: Table S2 — Comparison of the results of the present study for lipid levels with previous studies in European and Japanese populations. (DOC) [file pone.0082420.s002.doc]

**Table S2. Comparison of the results of the present study for lipid levels with previous studies in European and Japanese populations.**

| **Trait** | **SNP** | **Gene** | **Chr.** | **Position** | **Effect allele** | **European study ^a^** | | **Japanese study ^b^** | | **Present study ^c^** | |
| --- | --- | --- | --- | --- | --- | --- | --- | --- | --- | --- | --- |
|  |  |  |  |  |  | **Freq** | ***P* β ^d^** | **Freq** | ***P* β ^d^** | **Freq** | ***P* β ^d^** |
| **TC** | rs10903129 | *TMEM57* | 1 | 25641524 | G | 0.54 | 5.4×10^-10^ 0.061 | NA | NA | 0.26 | 0.48 -0.003 |
| **TC** | rs10889353 | *DOCK7* | 1 | 62890783 | C | 0.32 | 3.7×10^-12^ -0.079 | NA | NA | 0.20 | 9.39×10^-10^ -0.033 |
| **TC** | rs646776 | *CELSR2-PSRC1-SORT2* | 1 | 109620053 | G | 0.22 | 8.5×10^-22^ -0.128 | NA | NA | 0.04 | 1.64×10^-3^ 0.004 |
| **TC** | rs693 | *APOB* | 2 | 21085700 | G | 0.52 | 8.7×10^-23^ -0.096 | NA | NA | 0.95 | 0.63 -0.001 |
| **TC** | rs6756629 | *ABCG5* | 2 | 43918594 | G | 0.92 | 1.5×10^-11^ 0.145 | NA | NA | 0.99 | 0.50 -0.015 |
| **TC** | rs3846662 | *HMGCR* | 5 | 74688640 | G | 0.44 | 2.5×10^-19^ 0.092 | NA | NA | 0.53 | 1.47×10^-5^ 0.019 |
| **TC** | rs174570 | *FADS1-FADS2-FADS3* | 11 | 61353788 | G | 0.83 | 1.5×10^-10^ 0.088 | NA | NA | 0.40 | 0.31 0.003 |
| **TC** | rs2304130 | *NCAN-CLIP2-PBX4* | 19 | 19650528 | G | 0.07 | 2×10^-15^ -0.153 | NA | NA | 0.14 | 0.18 -0.015 |
| **TC** | rs2075650 | *TOMM40-APOE* | 19 | 50087459 | G | 0.15 | 2.9×10^-19^ 0.138 | NA | NA | 0.10 | 0.033 0.026 |
| **TG** | rs12130333 | *ANGPTL3-DOCK7-ATG4C* | 1 | 62964365 | T | 0.22 | 2×10^-8^ -0.11 | 0.02 | 0.283 -0.06 | 0.02 | 0.41 0.002 |
| **TG** | rs4846914 | *GALNT2* | 1 | 228362314 | G | 0.40 | 7×10^-15^ 0.08 | 0.74 | 7.33×10^-2^ -0.032 | 0.78 | 0.14 0.023 |
| **TG** | rs7557067 | *APOB* | 2 | 21061717 | G | 0.22 | 9×10^-12^ -0.08 | 0.66 | 0.816 -0.004 | 0.72 | 0.25 0.017 |
| **TG** | rs1260326 | *GCKR* | 2 | 27584444 | T | 0.45 | 2×10^-31^ 0.12 | 0.55 | 1.11×10^-11^ 0.101 | 0.44 | 3.26×10^-7^ 0.070 |
| **TG** | rs714052 | *MIXIPL* | 7 | 72502805 | G | 0.12 | 3×10^-15^ -0.16 | 0.11 | 3.3×10^-7^ -0.123 | 0.08 | 0.79 -0.004 |
| **TG** | rs17145738 | *BCL7B-TBL2-MLXIPL* | 7 | 72620810 | T | 0.13 | 7×10^-22^ -0.14 | NA | NA | 0.08 | 0.40 -0.017 |
| **TG** | rs7819412 | *XKR6-AMAC1L2* | 8 | 11082571 | G | 0.48 | 3×10^-8^ -0.04 | 0.91 | 0.175 0.042 | 0.93 | 0.23 0.031 |
| **TG** | rs12678919 | *LPL* | 8 | 19888502 | G | 0.10 | 2×10^-41^ -0.25 | 0.12 | 2.42×10^-13^ -0.166 | 0.14 | 3.04×10^-7^ -0.107 |
| **TG** | rs2954029 | *TRIB1* | 8 | 126560154 | T | 0.44 | 3×10^-19^ -0.11 | 0.53 | 2.91×10^-7^ -0.076 | 0.53 | 1.52×10^-4^ -0.052 |
| **TG** | rs174547 | *FADS1-FADS2-FADS3* | 11 | 61327359 | C | 0.33 | 2×10^-14^ 0.06 | 0.31 | 5.44×10^-3^ 0.043 | 0.40 | 0.15 0.021 |
| **TG** | rs964184 | *APOA1-APOC3-APOA4-APOA5* | 11 | 116154127 | G | 0.14 | 4×10^-62^ 0.3 | 0.27 | 1.67×10^-49^ 0.243 | 0.22 | 7.18×10^-16^ 0.130 |
| **TG** | rs17216525 | *NCAN-CLIP2-PBX4* | 19 | 19523220 | T | 0.07 | 4×10^-11^ -0.11 | 0.18 | 1.29×10^-3^ -0.073 | 0.09 | 0.04 -0.050 |
| **TG** | rs439401 | *TOMM40-APOE* | 19 | 50106291 | C | 0.09 | 1.8×10^-9^ 0.086 | 0.40 | 4.1×10^-4^ 0.054 | 0.46 | 8.78×10^-4^ 0.060 |
| **TG** | rs7679 | *PLTP* | 20 | 44009909 | C | 0.19 | 7×10^-11^ 0.07 | 0.01 | 0.664 -0.038 | 0.03 | 0.41 0.033 |
| **LDL** | rs11206510 | *PCSK9* | 1 | 55268627 | C | 0.19 | 4×10^-8^ -0.09 | 0.05 | 2.65×10^-2^ -0.098 | 0.05 | 0.13 -0.065 |
| **LDL** | rs646776 | *CELSR2-PSRC1-SORT2* | 1 | 109620053 | C | 0.24 | 3×10^-29^ -0.16 | 0.07 | 1.3×10^-3^ -0.116 | 0.04 | 1.23×10^-3^ -0.166 |
| **LDL** | rs693 | *APOB* | 2 | 21059688 | A | 0.48 | 1×10^-21^ 0.12 | 0.04 | 3.69×10^-3^ 0.132 | 0.06 | 0.66  0.015 |
| **LDL** | rs6544713 | *ABCG8* | 2 | 43927385 | T | 0.32 | 2×10^-20^ 0.15 | 0 | NA | 0 | NA |
| **LDL** | rs3846663 | *HMGCR* | 5 | 74691482 | T | 0.38 | 8×10^-12^ 0.07 | 0.53 | 2.5×10^-2^ 0.041 | 0.53 | 3.82×10^-6^ 0.089 |
| **LDL** | rs1501908 | *TIMD4-HAVCR1* | 5 | 156330747 | G | 0.37 | 1×10^-11^ -0.07 | 0.17 | 1.95×10^-2^ -0.066 | 0.29 | 4.17×10^-3^ -0.060 |
| **LDL** | rs2650000 | *HNF1A* | 12 | 119873345 | A | 0.36 | 2×10^-8^ 0.07 | 0.52 | 7.96×10^-3^ 0.048 | 0.56 | 0.47 0.014 |
| **LDL** | rs6511720 | *LDLR* | 19 | 11063306 | T | 0.1 | 2×10^-26^ -0.26 | 0 | NA | 0 | NA |
| **LDL** | rs10401969 | *NCAN-CLIP2-PBX4* | 19 | 19268718 | C | 0.06 | 2×10^-8^ -0.05 | 0.05 | 0.593 0.018 | 0.11 | 0.44 -0.022 |
| **LDL** | rs4420638 | *APOE-APOC1-APOC4-APOC2* | 19 | 50114786 | G | 0.16 | 4×10^-27^ 0.29 | 0.09 | 3.99×10^-5^ 0.132 | 0.11 | 0.05 0.072 |
| **LDL** | rs6102059 | *MAFB* | 20 | 38662198 | T | 0.32 | 4×10^-9^ -0.06 | 0.56 | 7.62×10^-3^ -0.049 | 0.54 | 0.04 -0.038 |
| **LDL** | rs6756629 | *ABCG5* | 2 | 43918594 | G | 0.92 | 2.6×10^-10^ 0.157 | NA | NA | 0.99 | 0.45 -0.071 |
| **LDL** | rs12670798 | *DNAH11* | 7 | 21573877 | G | 0.24 | 6.1×10^-9^ 0.089 | 0.48 | 0.887 0.003 | 0.53 | 0.76 0.005 |
| **HDL** | rs4846914 | *GALNT2* | 1 | 228362314 | G | 0.4 | 4×10^-8^ -0.05 | 0.74 | 2.91×10^-2^ -0.048 | 0.78 | 0.72 -0.003 |
| **HDL** | rs6754295 | *APOB* | 2 | 21059688 | C | 0.25 | 4.4×10^-8^ 0.068 | 0.71 | 0.355 -0.019 | 0.71 | 0.10 -0.012 |
| **HDL** | rs12678919 | *LPL* | 8 | 19888502 | G | 0.1 | 2×10^-34^ 0.23 | 0.18 | 9.41×10^-6^ 0.125 | 0.14 | 5.86×10^-11^ 0.053 |
| **HDL** | rs471364 | *TTC39B* | 9 | 15279578 | C | 0.12 | 3×10^-10^ -0.08 | 0.06 | 0.692 0.029 | 0.03 | 0.07 -0.030 |
| **HDL** | rs1883025 | *ABCA1* | 9 | 106704122 | T | 0.26 | 1×10^-9^ -0.08 | 0.26 | 2.24×10^-5^ -0.087 | 0.23 | 7.08×10^-4^ -0.023 |
| **HDL** | rs7395662 | *MADD-FOLH1* | 11 | 48475469 | G | 0.61 | 6×10^-11^ -0.073 | 0.54 | 0.427 -0.015 | 0.54 | 0.02 -0.011 |
| **HDL** | rs174547 | *FADS1-FADS2-FADS3* | 11 | 61327359 | C | 0.33 | 2×10^-12^ -0.09 | 0.31 | 0.654 -0.008 | 0.40 | 0.08 -0.012 |
| **HDL** | rs964184 | *APOA1-APOC3-APOA4-APOA5* | 11 | 116154127 | G | 0.14 | 1×10^-12^ -0.17 | 0.26 | 2.41×10^-7^ -0.106 | 0.22 | 0.05 -0.016 |
| **HDL** | rs2338104 | *MMAB-MVK* | 12 | 108379551 | C | 0.45 | 1×10^-10^ -0.07 | 0.72 | 0.188 -0.029 | 0.62 | 0.06 -0.012 |
| **HDL** | rs10468017 | *LIPC* | 15 | 56465804 | T | 0.3 | 8×10^-23^ 0.1 | 0.2 | 4.14×10^-4^ 0.079 | 0.18 | 0.29 -0.001 |
| **HDL** | rs1800775 | *CETP* | 16 | 55552737 | C | 0.51 | 1×10^-73^ -0.18 | 0.46 | 1.66×10^-7^ -0.096 | 0.49 | 8.94×10^-3^ -0.020 |
| **HDL** | rs2271293 | *LCAT* | 16 | 66459571 | A | 0.11 | 9×10^-13^ 0.07 | 0.03 | 0.247 0.05 | 0.03 | 0.05 -0.043 |
| **HDL** | rs4939883 | *LIPG* | 18 | 45421212 | T | 0.17 | 7×10^-15^ -0.14 | 0.22 | 8.18×10^-2^ -0.039 | 0.18 | 0.17 -0.005 |
| **HDL** | rs2967605 | *ANGPTL4* | 19 | 8375738 | T | 0.16 | 1×10^-8^ -0.12 | 0.53 | 0.544 -0.011 | 0.60 | 0.87 -0.007 |
| **HDL** | rs1800961 | *HNF4A* | 20 | 42475778 | T | 0.03 | 8×10^-10^ -0.19 | 0.01 | 0.191 -0.123 | 0.01 | 0.05 -0.122 |
| **HDL** | rs7679 | *PLTP* | 20 | 44009909 | C | 0.19 | 4×10^-9^ -0.07 | 0.01 | 0.546 0.067 | 0.03 | 0.86 -0.006 |

Chr., chromosome; Freq, frequency; NA: data not available.

^a^ The *P* values in European population were cited from Reference (4, 5, 6).

^b^ The *P* values in Japanese population were cited from Reference (7).

^c^ The *P* values in Han Chinese population were from meta-analysis of two GWAS (DFTJ-cohort and FAMHES) in discovery stage in present study.
