# Supplementary material for: A Genome Wide Association Study Identifies Common Variants Associated with Lipid Levels in the Chinese Population
Source: PLoS One. 2013 Dec 30;8(12):e82420. doi: 10.1371/journal.pone.0082420 (PMC3875415; doi:10.1371/journal.pone.0082420)
Supplement: Figure S1 — Q-Q plots for QTL analyses. The horizontal axis shows -log10 transformed expected P values, while the vertical axis indicates -log10 transformed observed P values. The genomic inflation factor λ for each analysis is shown below each graph. Black line, all test statistics; red line, 35 previously reported loci (Table S2) excluded. (DOC) [file pone.0082420.s003.doc]

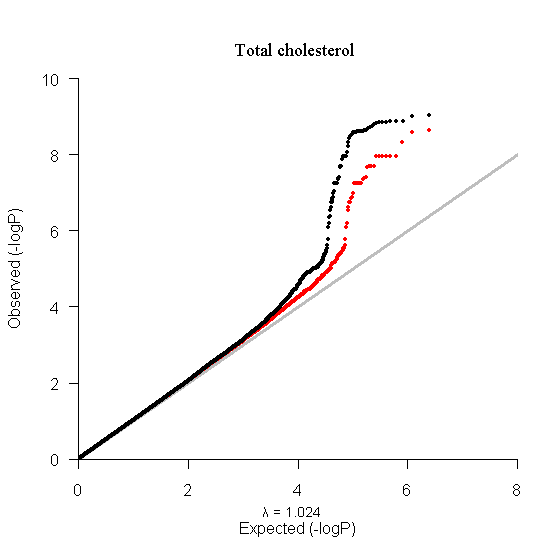

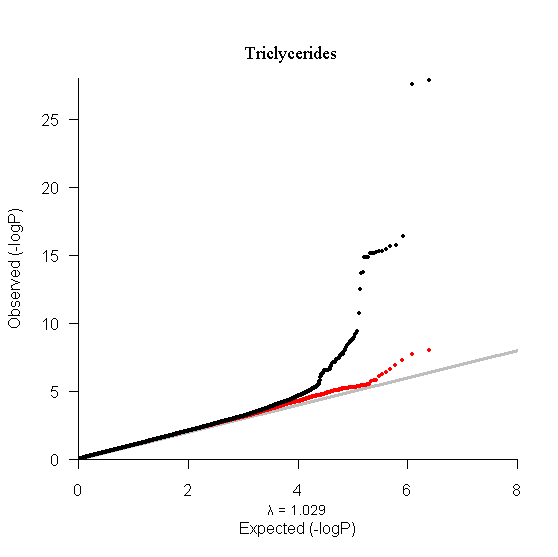


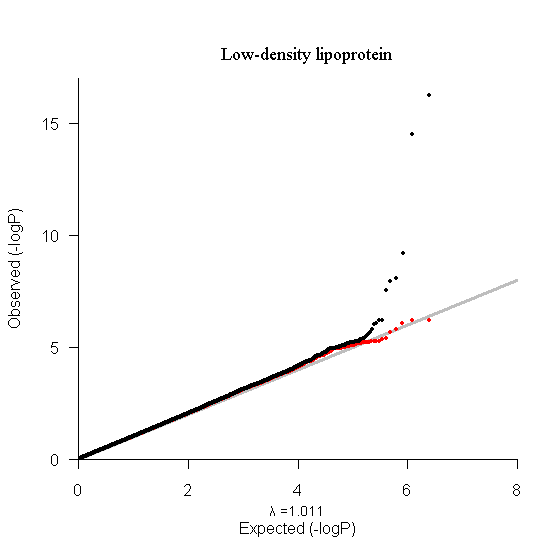

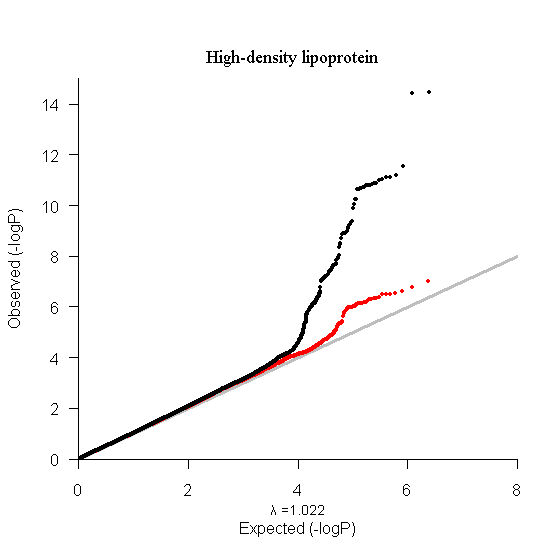


**Figure S1. Q-Q plots for QTL analyses.** The horizontal axis shows -log10 transformed expected *P* values, while the vertical axis indicates -log10 transformed observed *P* values. The genomic inflation factor λ for each analysis is shown below each graph. Black line, all test statistics; red line, 35 previously reported loci (Table S2) excluded.
