# Supplementary material for: A Genome Wide Association Study Identifies Common Variants Associated with Lipid Levels in the Chinese Population
Source: PLoS One. 2013 Dec 30;8(12):e82420. doi: 10.1371/journal.pone.0082420 (PMC3875415; doi:10.1371/journal.pone.0082420)
Supplement: Figure S2 — Regional plots of associated loci with lipid levels. The horizontal axis shows the chromosomal positions in the NCBI build 36 genome sequence. (DOC) [file pone.0082420.s004.doc]

TC


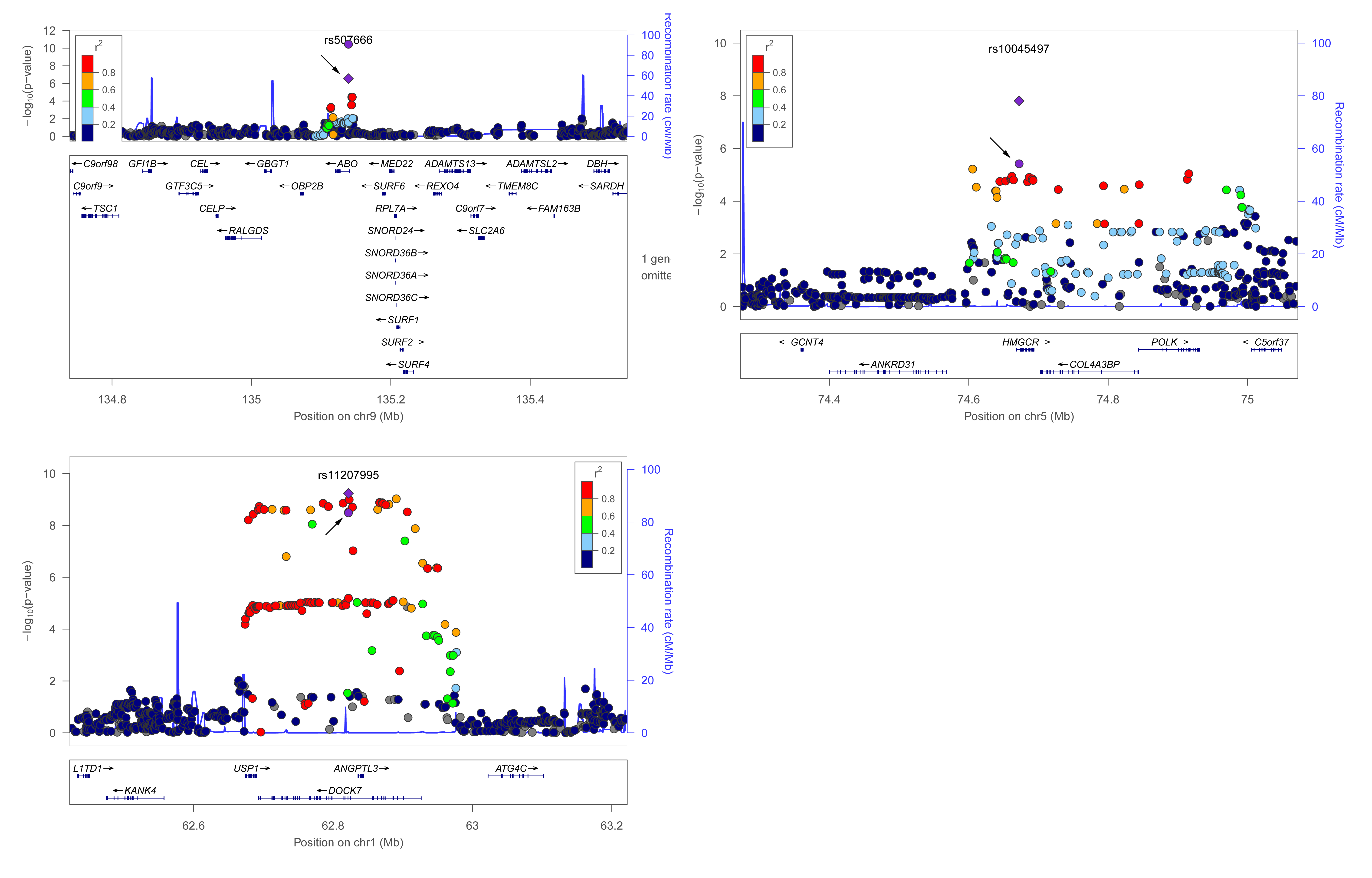


TG


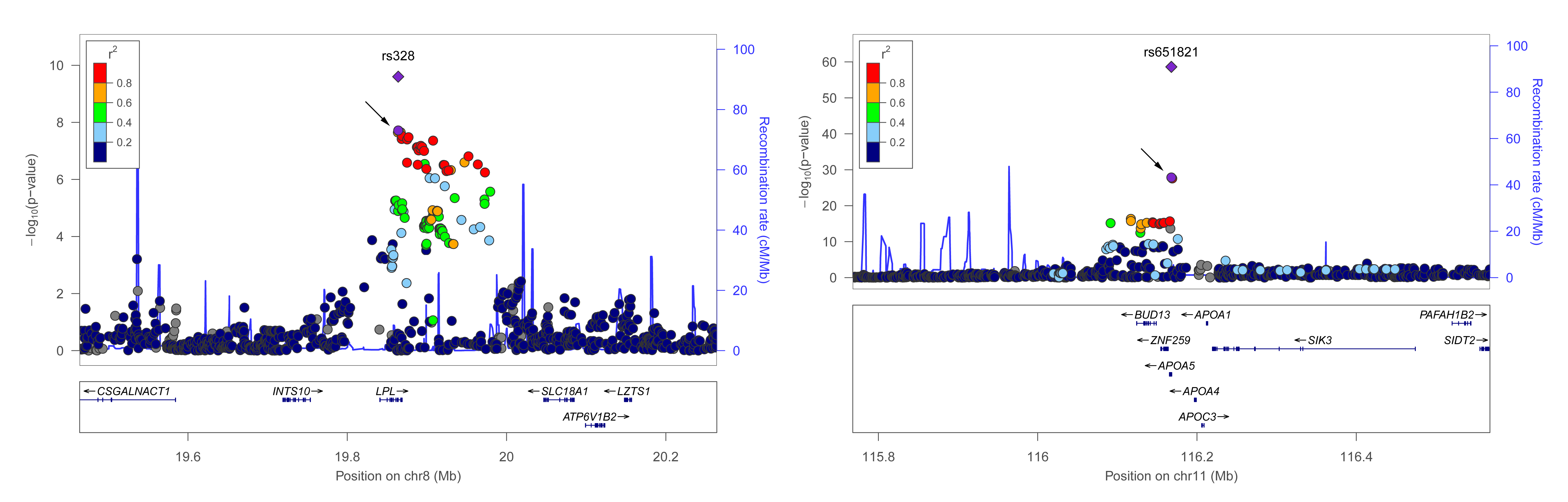


LDL


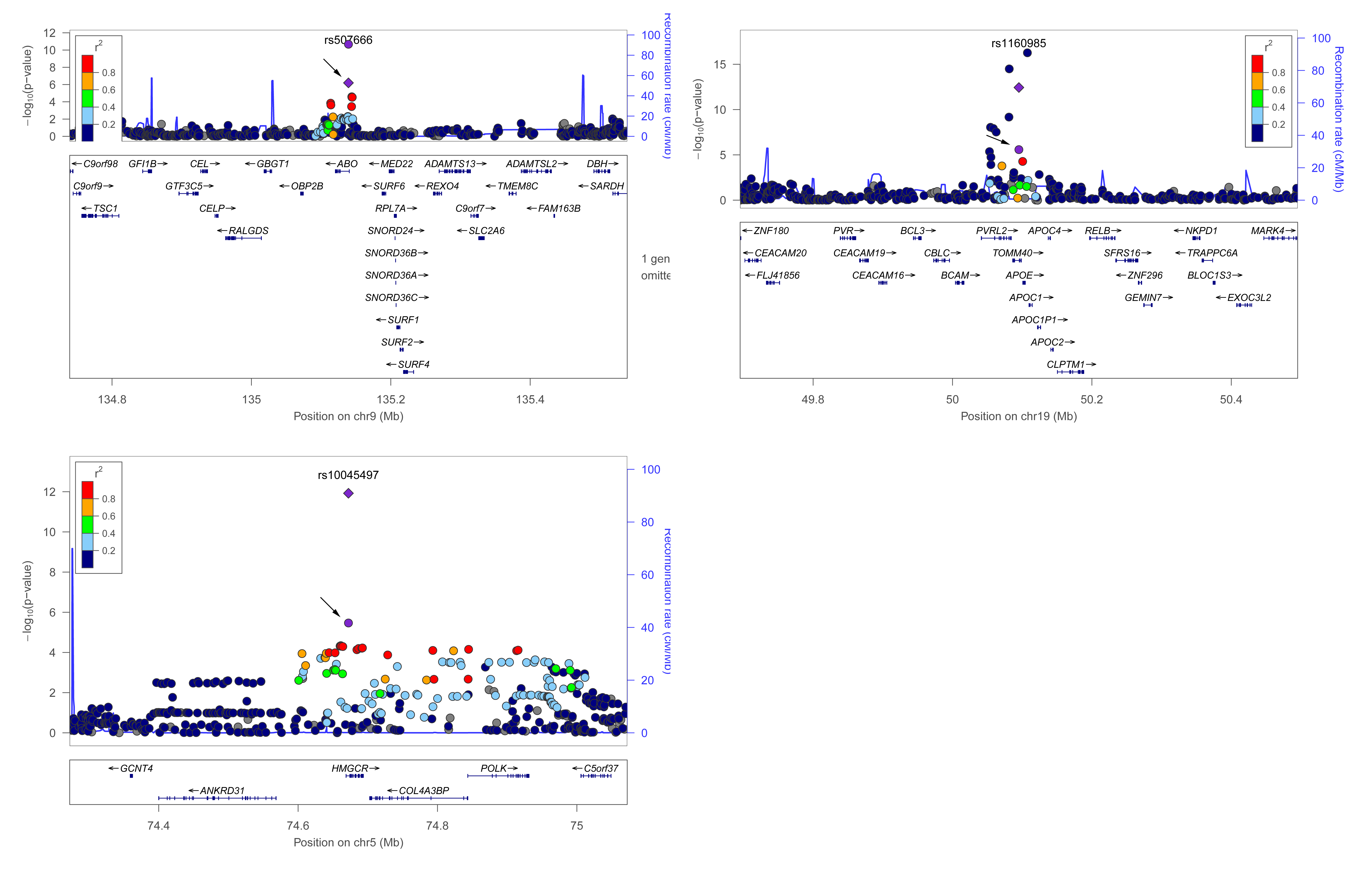


HDL


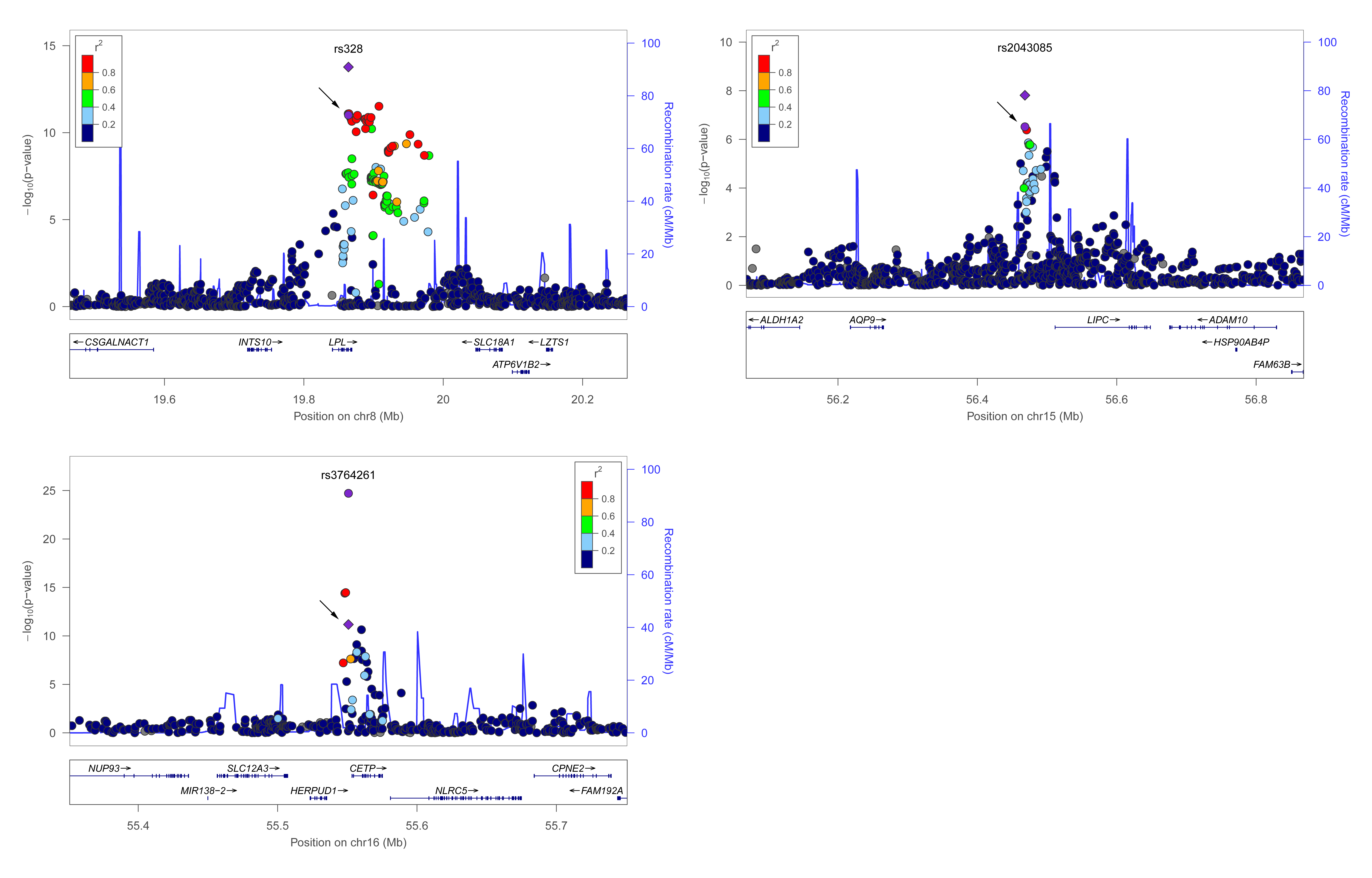


**Figure S2. Regional plots of associated loci with lipid levels.** The horizontal axis shows the chromosomal positions in the NCBI build 36 genome sequence.
