# Supplementary material for: A Genome Wide Association Study Identifies Common Variants Associated with Lipid Levels in the Chinese Population
Source: PLoS One. 2013 Dec 30;8(12):e82420. doi: 10.1371/journal.pone.0082420 (PMC3875415; doi:10.1371/journal.pone.0082420)
Supplement: Figure S3 — Principal component analysis (PCA) plot of samples in the current GWAS. The plot presents the top two eigenvectors identified by principal component analysis using Eigenstrat software. (DOC) [file pone.0082420.s005.doc]

**
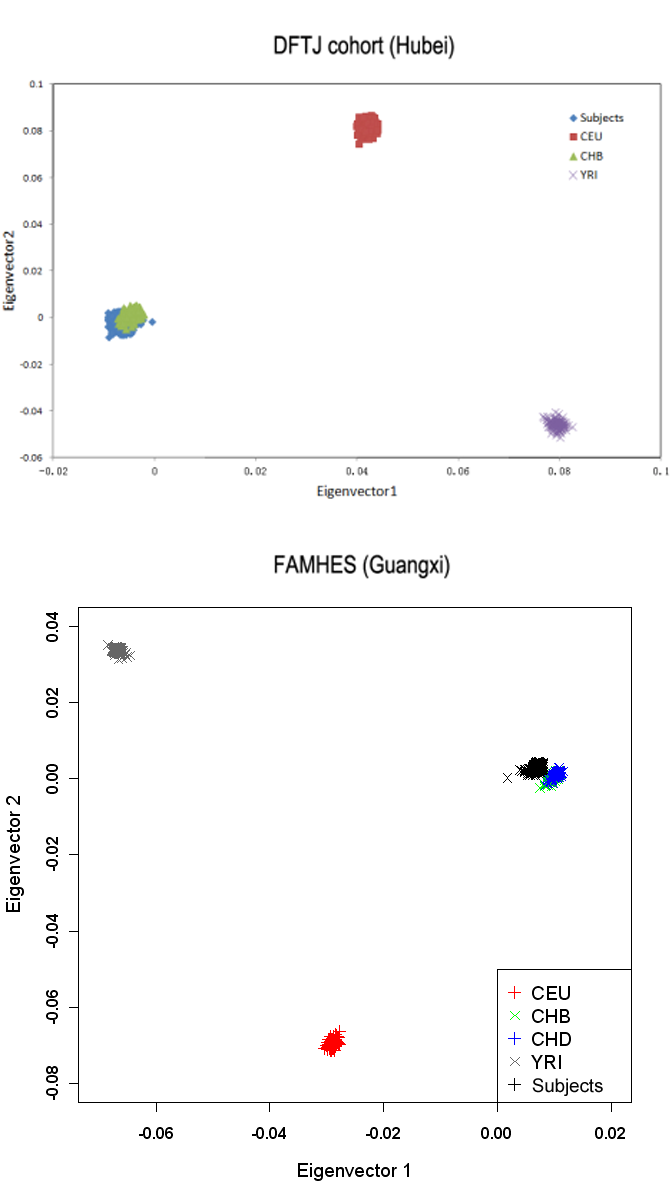
**

**Figure S3. Principal component analysis (PCA) plot of samples in the current GWAS.** The plot presents the top two eigenvectors identified by principal component analysis using Eigenstrat software.
